# Microglia regulate hippocampal neurogenesis during chronic neurodegeneration

**DOI:** 10.1016/j.bbi.2015.11.001

**Published:** 2016-07

**Authors:** Chiara De Lucia, Adeline Rinchon, Adrian Olmos-Alonso, Kristoffer Riecken, Boris Fehse, Delphine Boche, V. Hugh Perry, Diego Gomez-Nicola

**Affiliations:** a Centre for Biological Sciences, University of Southampton, Southampton, United Kingdom; b Research Department Cell and Gene Therapy, Clinic for Stem Cell Transplantation, University Medical Centre (UMC) Hamburg-Eppendorf, Hamburg, Germany; c Clinical Neurosciences, Clinical and Experimental Sciences, Faculty of Medicine, University of Southampton, United Kingdom

**Keywords:** Dentate gyrus, ME7, CSF1R, GW2580, TGFb

## Abstract

Neurogenesis is altered in neurodegenerative disorders, partly regulated by inflammatory factors. We have investigated whether microglia, the innate immune brain cells, regulate hippocampal neurogenesis in neurodegeneration. Using the ME7 model of prion disease we applied gain- or loss-of CSF1R function, as means to stimulate or inhibit microglial proliferation, respectively, to dissect the contribution of these cells to neurogenesis. We found that increased hippocampal neurogenesis correlates with the expansion of the microglia population. The selective inhibition of microglial proliferation caused a reduction in neurogenesis and a restoration of normal neuronal differentiation, supporting a pro-neurogenic role for microglia. Using a gene screening strategy, we identified TGFβ as a molecule controlling the microglial pro-neurogenic response in chronic neurodegeneration, supported by loss-of-function mechanistic experiments.

By the selective targeting of microglial proliferation we have been able to uncover a pro-neurogenic role for microglia in chronic neurodegeneration, suggesting promising therapeutic targets to normalise the neurogenic niche during neurodegeneration.

## Highlights

• Microglia are proneurogenic in a model of prion disease.
• Blocking the expansion of microglia prevents the aberrant differentiation of newborn neurons.
• TGFβ dominates the proneurogenic actions of microglia during chronic neurodegeneration.

## 1 Introduction

Mammalian adult hippocampal neurogenesis is an active process in the healthy brain and is modulated in response to brain injury and neurodegeneration (Parent, 2003). However, the regulation of hippocampal neurogenesis in chronic neurodegenerative diseases is unclear, as the animal models for multi-factorial diseases such as Alzheimer’s disease (AD) often fail to reproduce the entire pathology, adding more variability to an already heterogeneous landscape (Kokjohn and Roher, 2009). Thus, there is a clear need for a better understanding of neurogenesis during chronic neurodegeneration, arising from experimental models that recapitulate the human pathophysiology. In this line, models of prion disease have been used to mimic chronic neurodegeneration, as they are reproducible and recapitulate phenotypes present in several neurodegenerative diseases, such as microglial activation, misfolded protein deposition and neurodegeneration, making them an excellent model to study some of the mechanisms involved in neurodegeneration and neurogenesis (Gomez-Nicola and Perry, 2014a, Gomez-Nicola et al., 2014c). Although models of prion disease differ from models of AD-like pathology in their duration (5 months vs. 6–18 months), severity (models of AD-like pathology show no neuronal death while prion disease has extensive neurodegeneration) and induction (models of prion disease are inducible vs. AD-like pathology are transgenic), they better resemble the pathophysiology of human chronic neurodegeneration. We recently described an aberrant over-activation of the neurogenic cascade during chronic neurodegeneration in models of prion disease, which led to an effective compensatory replacement of degenerating granule cells, and correlates with findings from human post-mortem tissue (Gomez-Nicola et al., 2014c). However, the molecular determinants of this process are still to be unravelled.

A key component in the interplay between neurodegeneration and neurogenesis is inflammation (Amor and Woodroofe, 2014). Inflammation is a common factor in many neurodegenerative diseases, triggered by neuronal damage, protein misfolding and amyloid deposition. Although microglia are the main inflammatory cells in the CNS, their exact role in regulating neurogenesis remains largely unknown in health and even more elusive in disease. However, neuroinflammation has been shown to alter neurogenesis in various conditions. By interacting with neuronal precursors, microglia can increase neurogenesis after environmental enrichment or decrease it in response to ageing (Kohman et al., 2013, Ziv et al., 2006). The impact of microglia on neurogenesis seems to depend on their activation profile, with pro-inflammatory states impairing neurogenesis and anti-inflammatory states supporting it (Butovsky et al., 2006, Kohman et al., 2013). Microglial activation following LPS decreases neuronal survival (Ekdahl et al., 2003, Monje et al., 2003, Valero et al., 2014) and inhibits neural stem cells (NSCs) proliferation (Fujioka and Akema, 2010) and affects cell integration into existing neural circuits (Jakubs et al., 2008). On the other hand, activation of microglia with an anti-inflammatory molecule, such as TGFβ, increases NSCs proliferation (Mathieu et al., 2010). The neurogenic impairment seen after LPS injection can be rescued by treatment with minocycline or anti-inflammatory drugs, suggesting a microglia-dependent mechanism (Ekdahl et al., 2003, Monje et al., 2003). Together, these studies suggest a key role for microglia in regulating neurogenesis in disease. A better understanding of how neuroinflammation contributes to neurogenesis is therefore crucial to better discern the self-repairing potential of the brain (Miller and Gomez-Nicola, 2014).

In our previous studies, we identified the crucial role of colony-stimulating factor 1 receptor (CSF1R) for the expansion and inflammatory activation of the microglial population in prion disease (Gomez-Nicola et al., 2013, Gomez-Nicola et al., 2014b). The activation of CSF1R by its ligands CSF1 and IL-34 is key for the maintenance of microglial proliferation and survival in both health and disease (Gomez-Nicola and Perry, 2014a). Inhibition of CSF1R activation with a selective tyrosine kinase inhibitor (GW2580) is an effective way to arrest the expansion of microglia and to drive a shift towards an anti-inflammatory profile (Gomez-Nicola et al., 2013), providing us with a valuable tool to target the contribution of microglia to neurogenesis.

In summary, the role of microglia in the regulation of neurogenesis in chronic neurodegeneration needs to be explored in detail. Experimental models of prion disease are predictable systems to study the interaction of microglia and neurogenesis, as previously described by our group (Gomez-Nicola et al., 2013, Gomez-Nicola et al., 2014c). In particular, we hypothesise that the control of microglial numbers, by means of up- or down-regulating their proliferation (gain- or loss-of-activity of CSF1R), will help dissecting the contribution of these cells to hippocampal neurogenesis in prion disease, and further identify any molecular determinants of this response. Our findings support a pro-neurogenic role of microglia in prion disease and highlight the crucial role of TGFβ in regulating this process. Our results link neuroinflammation and neurogenesis during chronic neurodegeneration, and open new avenues to explore the therapeutic value of immunomodulatory strategies to control the self-repairing potential of the brain.

## 2 Materials and methods

### 2.1 Animal tissue and ethical licence

C57BL/6J (Harlan) and c-fms-EGFP mice (macgreen) (Sasmono et al., 2003) were bred and maintained at the University of Southampton. Mice expressing EGFP under the promoter of c-fms (CSF1R) are characterised by the expression of green fluorescence in microglial cells and other macrophages. Male or female mice were housed in groups of 4–10, under a 12-h light/12-h dark cycle at 21 °C, with food and water ad libitum. Prion disease was induced under anaesthesia with a ketamine/rompun mixture (85 and 13 mg/kg respectively), and injection of 1 μl of either ME7-derived (ME7-animals; model of prion disease; 10% w/v) or normal brain homogenate (NBH-animals), injected stereotaxically and bilaterally in the brain at the coordinates from bregma: anteroposterior, −2.0 mm; lateral, −1.7 mm; depth, 1.6 mm. All procedures were performed in accordance with U.K. Home Office licensing.

### 2.2 Gain/loss of activity of CSF1R

For gain of function experiments, NBH (control) and ME7 (prion) macgreen mice were treated at 12 weeks post-injection with 50 ng of recombinant murine CSF1 (Merck Chemicals; *n* = 4), murine IL34 (R&D Systems; *n* = 4) or saline (vehicle; *n* = 4) by stereotactic injection in the dorsal hippocampus (CA1 field; anteroposterior, −2.0 mm; lateral, −1.7 mm; depth, 1.6 mm) with a Hamilton syringe. Mice received 5 injections of i.p. BrdU (Sigma–Aldrich; 7.5 mg/ml, 0.1 ml/10 g weight in sterile saline), before the end of the experiment (+1 week), as previously described (Gomez-Nicola et al., 2013).

Inhibition of the tyrosine kinase activity of CSF1R was achieved by the administration of GW2580, as previously described (Gomez-Nicola et al., 2013). GW2580 (LC Laboratories) was suspended in 0.5% hydroxypropylmethylcellulose and 0.1% Tween 80 and was dosed by oral gavage, using gavaging needles, at 0.2 ml per mouse (75 mg/kg), daily for 4 consecutive weeks (from 14th to 18th week post-injection), to NBH and ME7 mice (*n* = 8), using the vehicle as control (*n* = 8). Mice weight was monitored during the experiment. Mice received 2 daily injections of i.p. BrdU (Sigma–Aldrich; 7.5 mg/ml, 0.1 ml/10 g weight in sterile saline), before the end of the experiment (18th week).

### 2.3 Immunohistochemistry

Mice perfusion, tissue processing and immunohistochemical analysis were carried out as previously described (Gomez-Nicola et al., 2013, Gomez-Nicola et al., 2014c). The general immunohistochemistry protocol was modified for the detection of BrdU, adding a DNA denaturation step with 2 N HCl (30 min, 37C) before blocking. The following primary antibodies were used: goat anti-DCX (Doublecortin; Santa Cruz Biotechnologies), mouse anti-BrdU (DSHB), mouse anti-NeuN (Millipore), rabbit anti-GFAP (Glial Fibrillary Acidic Protein; Dako) and rabbit anti-TGFβ (Abcam). Following primary antibody incubation, the sections were washed and incubated with the appropriate biotinylated secondary antibody (Vector Labs) followed by ABC amplification (Vector Labs), and/or with the appropriate Alexa 405, 488 or 568 conjugated secondary antibody or streptavidin (Molecular Probes). For co-labelling in brightfield, following primary antibody, sections were incubated with a biotinylated secondary antibody (Vector Labs) followed by ABC amplification (Vector Labs), and the ImmPRESS-AP (alkaline phosphatase) Polymer Detection Kit. For light microscopy, the sections were visualised using diaminobenzidine (DAB) precipitation and/or BCIP/NBT AP reaction, in a Leica CTR 5000 microscope, coupled to a Leica DFC300FX microscope camera. After immunofluorescence labelling, nuclei were visualised by DAPI staining and the sections were mounted with Mowiol/DABCO (Sigma–Aldrich) mixture. The sections were visualised on a Leica TCS-SP5 confocal system, coupled to a Leica CTR6500 microscope.

### 2.4 Tracing of hippocampal neurogenesis with γ-retroviral vectors

The delivery of Eco-SFFV mCherry γ-retroviral vectors was used to trace hippocampal neurogenesis in NBH or ME7 macgreen mice treated with GW2580 or vehicle. Viral vector design and production was performed as previously described (Gomez-Nicola et al., 2014a). At 14 weeks post-induction, mice were anaesthetised with a ketamine/rompun mixture (85 and 13 mg/kg), and 1 μl (10^9^ particles/ml) of the viral particles were injected stereotaxically and bilaterally in the dentate gyrus (hilus) of the hippocampus at each of the following coordinates from bregma: anteroposterior, −2.0 mm; lateral, ±1.3 mm; depth, −2 mm and anteroposterior, −1.5 mm; lateral, ±0.8 mm; depth, −2 mm.

Mouse perfusion, tissue processing and analysis were performed as previously described, at 18 weeks post-induction (4 weeks post tracing) (Gomez-Nicola et al., 2014a, Gomez-Nicola et al., 2014c). Coronal sections were cut with a vibrating microtome from paraformaldehyde-fixed brains (50 μm). After repeated rinses with PBS, and counterstaining with DAPI (Sigma–Aldrich), free-floating sections were mounted onto glass slides and coverslipped with a Mowiol/DABCO (Sigma–Aldrich) mixture. Sections were visualised on a Leica TCS-SP5 confocal system, coupled to a Leica CTR6500 microscope. Confocal stacks were acquired using 1 μm Z steps to cover the whole morphology of the labelled cells and their processes. Cells within the dorsal and ventral blade of the dorsal dentate gyrus were analysed in this study.

Sholl analysis and dendrite morphometric analysis was performed on 3D reconstructions of confocal Z-stacks of mCherry-traced newborn neurons (Gomez-Nicola et al., 2014c, Sholl, 1953). Briefly, the number of crossings for concentric circles of given radii centred at the tree stem (Sholl number) was calculated. Starting radius and radius step size were set each at 10 μm up to 300 μm. All quantifications were performed with the help of the image analysis software ImageJ, additionally using NeuronJ and Sholl Analysis plug-ins.

### 2.5 Candidate gene expression screening

To pinpoint potential microglia-derived molecular candidates regulating hippocampal neurogenesis in prion disease, we performed a selection strategy using available expression databases and qPCR analysis. First, a literature review was performed to select the most important molecular pathways previously reported to link inflammation with neurogenesis. For these pathways, we shortlisted the main molecular mediators defining the pathway function to progress in the screening (Sup File1). A list of 29 candidate genes were identified and their mRNA expression levels checked in the ‘Prion Disease Database (Gehlenborg et al., 2009, Hwang et al., 2009), a microarray database providing mRNA expression data of whole brain homogenates in prion disease, and a microarray database previously published by our group, from hippocampal samples of ME7 and NHB mice (Lunnon et al., 2011). Genes showing significant and consistent change were further analysed by qPCR in samples microdissected from the dentate gyrus (Gomez-Nicola et al., 2014c) (NBH and ME7; 12 and 20 weeks post-induction) to determine if their alterations were specific to the neurogenic niche and their time-course. Fresh frozen brain sections were cut with a cryostat, to then dissect the DG area of the hippocampus, under a dissecting microscope. Samples were homogenised in Trizol reagent (Invitrogen), following the manufacturer instructions to isolate RNA, as previously described (Gomez-Nicola et al., 2014b). The isolated RNA was quantified (Nanodrop, Thermo Scientific) and retro-transcribed to cDNA with the iScript RT kit (Bio-Rad), after checking its integrity by electrophoresis in a 2% agarose gel. cDNA libraries were analyzed by RT-PCR using the iTaqTM Universal SYBR® Green supermix (Bio-Rad) and the custom designed gene-specific primers (Sigma–Aldrich; Sup File2). The amplification of DNA was detected by SYBRgreen fluorescence. Quality of the primers and the PCR reaction were evaluated by electrophoresis in a 1.5% agarose gel, checking the PCR product size. Data were analyzed using the ΔΔCt method with Primer Opticon 3 software, using GAPDH as a housekeeping gene.

Genes showing significant up- or down-regulation in ME7 compared to NBH were shortlisted for the last screen, completed in DG microdissected samples from NBH, ME7+vehicle and ME7+GW2580 mice, in order to evaluate the effect of the microglial component on the expression of these candidate regulators of neurogenesis, as GW2580 has proven efficacy in selectively inhibiting microglial proliferation and activation in prion disease (Gomez-Nicola et al., 2013). qPCR analysis was completed as described above, in order to identify mediators showing a expression change coincident with the change observed in neurogenesis when treating with GW2580 (number of DCX^+^ cells).

### 2.6 Gene targeting of TGFβ expression

We used adenoviral vectors to target the activity of TGFβ, by driving the overexpression of Decorin, a natural inhibitor of TGFβ, as detailed before (Boche et al., 2006). Briefly, the AdDcn adenovirus expressing human decorin cDNA and the AdDL70-3 adenovirus (control) were used to inhibit TGFβ and as an inactive control, respectively. NBH or ME7 mice underwent bilateral intra-hippocampal injections with 1 μl of 10^5^ pfu/μl of adenoviral solution 13 weeks after post-disease induction. The animals were sacrificed 12 days after adenoviral injection and the tissues were prepared for immunohistochemistry and RNA isolation, as detailed above. Details on the design of the adenoviral constructs can be found in Bett et al., 1994. The effectiveness of the AdDcn in inhibiting TGFβ production is documented in the literature (Al Haj Zen et al., 2003, Zhao et al., 1999). TGFβ expression levels were analysed in animals treated with AdDcn or AdDL70-3 adenovirus. Formalin-fixed paraffin wax embedded hippocampi were dissected and RNA isolation was performed with the ‘’RecoverAll™ Total Nucleic Acid Isolation Kit for FFPE (Life technologies). Reverse transcription and RT-PCR analysis was performed as described above.

### 2.7 Quantification and image analysis

The quantification of antigen positive cells was performed in DG, SGL (subgranular layer), hilus or ML (molecular layer) (*n* = 4 fields/mouse, *n* = 4–6 mice/group) after DAB immunohistochemistry. Data were represented as number of positive cells/mm^2^. To investigate microglial density surrounding DCX^+^ clusters, areas with low (0–2 adjacent DCX^+^ cells) or high (2 or more adjacent DCX^+^ positive cells) density of immature neurons were identified and the number of Iba1^+^ cells/mm^2^ was calculated within them. For co-localisation studies, the total number of Iba1^+^ cells/mm^2^ and the number of Iba1^+^/TGFβ^+^ cells/mm^2^ were quantified to obtain a percentage of co-localisation. All quantifications were performed with the help of the image analysis software ImageJ.

### 2.8 Statistical analysis

Data were expressed as mean ± SEM and analysed with the GraphPad Prism 5 software package (GraphPad Software). For all data sets, normality and homoscedasticity assumptions were reached, validating the application of the one-way or two-way ANOVA, followed by the Tukey post-hoc test for multiple comparisons. The within-subjects variable used was brain region. The between-subjects variables used were: disease (NBH vs. ME7) and treatment. Differences were considered significant for *p* < 0.05.

## 3 Results

### 3.1 Microglia cells expand homogeneously at the different layers of the dentate gyrus during prion disease

**Fig. 1:**
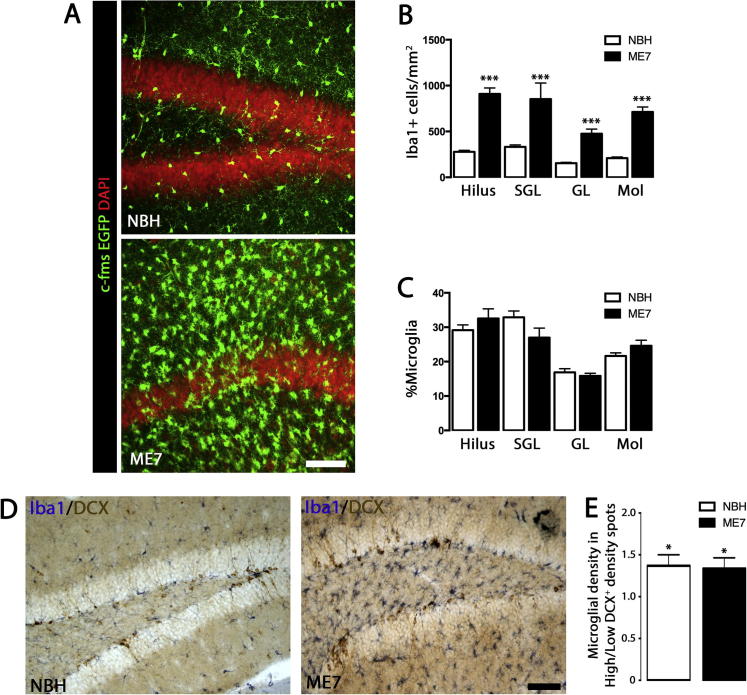
Increased microglisis in the dentate gyrus of prion-diseased mice. (A–C) Quantification of the density (B) or relative sub-regional distribution (C) of microglial cells (Iba1^+^) in the dentate gyrus (DG) of prion-diseased mice (ME7; black bars) or NBH controls (normal brain homogenate, open bars). Representative images from c-fms EGFP NBH and ME7 mice shown in A (nuclei shown in red, DAPI). (B) Quantified data expressed as mean ± SEM of the number of Iba1^+^ cells/mm^2^. (C) Quantified data expressed as mean ± SEM of the % of Iba1^+^ cells at a specific layer from the total population of Iba1^+^ at the dentate gyrus (DG). (D, E) Analysis of the cell-contact interaction of microglial cells (Iba1^+^) with neural progenitor cells (NPCs, DCX^+^) at the DG of ME7 or NBH mice. Microglial density was measured at high- or low-density DCX^+^ clusters. (E) Quantified data expressed as mean ± SEM of the number of Iba1^+^ cells/mm^2^ at high/low density DCX^+^ clusters. Statistical differences: ^*^p < 0.05 (vs. matching NBH area), ^***^p < 0.001 (vs. matching NBH area). Data were analysed with a two-way ANOVA, showing statistical comparisons arising from a post-hoc Tukey test (*n* = 4–6). Scale bar in (A, D) 100 μm.

Following the recent observation of microglial proliferation in the dentate gyrus (DG) of prion diseased mice (Gomez-Nicola et al., 2014c), we studied the sub-regional microglial expansion in the different layers of the DG in the ME7 model of prion disease, to determine whether the dynamics of the microglial population was locally associated with the neurogenic niche (subgranular layer; SGL) (Fig. 1). We quantified the density of microglia in the hilus, SGL, granular layer (GL) and molecular layer (Mol) of the DG in ME7 mice compared with NBH controls, and observed significant increases in all the sub-regions analysed (Fig. 1A, B). We calculated the percentage distribution throughout the layers, to test whether microglia were expanding preferentially in some sub-regions, and found no significant change in microglial percentage distribution between ME7 and NBH mice (Fig. 1C). These results support a homogeneous expansion in the microglial population in the DG during ME7.

We further tested the hypothesis of a localised cell-contact interaction of microglial cells with neural progenitor cells (NPCs; DCX^+^) in the ME7 model. We previously reported that DCX^+^ cells form high density clusters in this model (Gomez-Nicola et al., 2014c), so we analysed if microglial numbers correlate with the expansion of DCX^+^ cells, grouped as high- or low-density clusters (Fig. 1D, E). The increased density of DCX^+^ cells correlated with the increased density of microglial cells, being this association equal in both ME7 and NBH mice (1.3 fold increase) (Fig. 1D, E).

Our results show that, although the microglial population expands in the DG of prion diseased mice, there is no clear physical association with the neurogenic niche, suggesting that a potential neuromodulatory action of microglia shall be mediated through diffusible factors.

### 3.2 The inhibition of microglial proliferation during prion disease reveals a pro-neurogenic activity of microglia

**Fig. 2:**
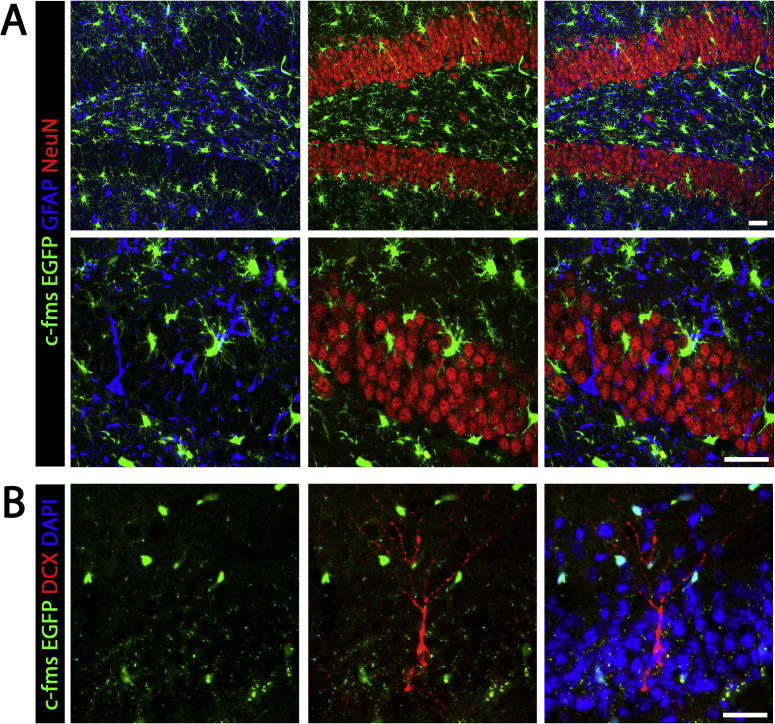
CSF1R is not expressed by neurons, astrocytes or NPCs. (A) Immunohistochemical analysis, coupled to confocal microscopy, of the localisation of EGFP (driven by the c-fms (CSF1R) promoter in macgreen mice) in astrocytes (GFAP^+^, blue) or neurons (NeuN^+^, red) in the hippocampus of ME7 mice. (B) Immunohistochemical analysis, coupled to confocal microscopy, of the localisation of EGFP (driven by the c-fms (CSF1R) promoter in macgreen mice) in neural progenitor cells (NPCs, DCX^+^; red) at the dentate gyrus (DG) of ME7 mice. Nuclei are stained in blue. Scale bar in (A, B) 50 μm.

To investigate the potential regulatory activity of microglia on neurogenesis, we took advantage of their dependence on CSF1R signalling in the control of the expansion of this population (Gomez-Nicola et al., 2013). Thus, by either over-activating or inhibiting the activity of this receptor we can control microglial numbers in ME7 mice and analyse possible downstream effects of microglia on neurogenesis. We have taken advantage of the fact that the expression of EGFP in macgreen mice is driven by the CSF1R promoter (c-fms), making it an optimal reporter mouse for analysing the specificity of CSF1R expression (Sasmono et al., 2003). Using immunofluorescence and confocal microscopy we found no evidence of expression of CSF1R in neurons (NeuN^+^; Fig. 2A), astrocytes (GFAP^+^; Fig. 2A) or NPCs (DCX^+^; Fig. 2B), supporting that CSF1R expression is exclusive of microglia (Erblich et al., 2011, Gomez-Nicola et al., 2013, Sasmono et al., 2003).

**Fig. 3:**
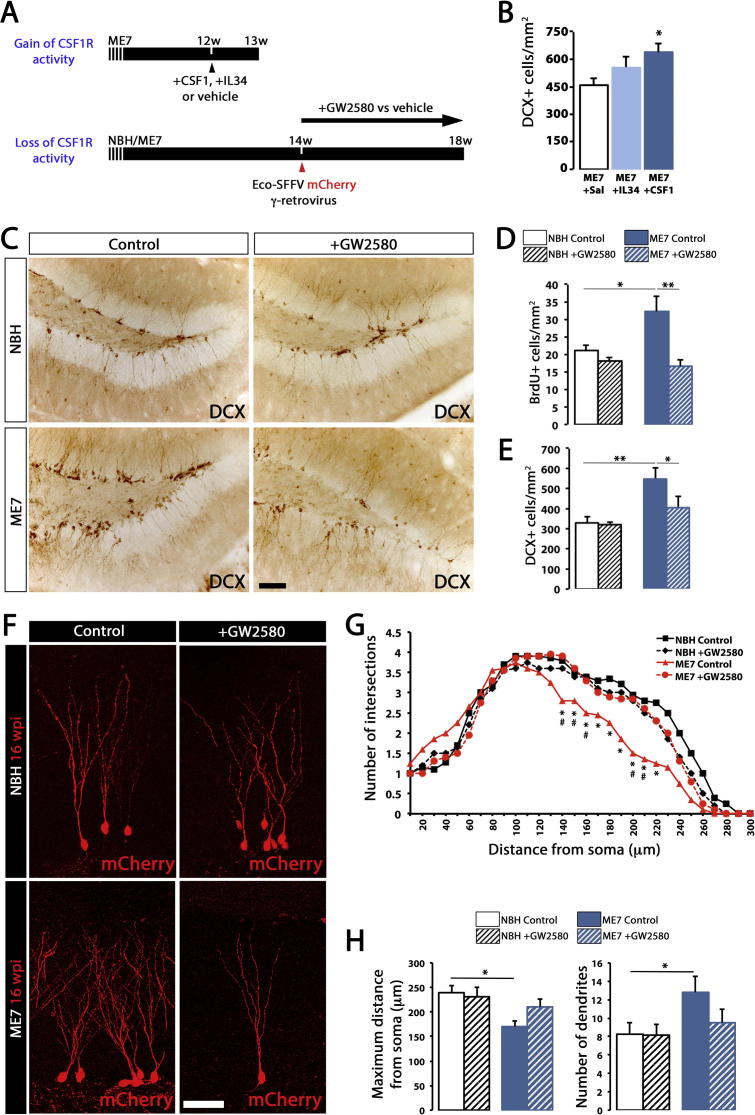
The gain- or loss-of-activity of CSF1R reveals a pro-neurogenic role of microglia in prion disease. (A) Gain of activity of CSF1R was achieved by the intrahippocampal administration of recombinant CSF1, IL34 or vehicle in ME7 mice, while loss of activity of CSF1R achieved by the prolonged oral dosing of GW2580, as previously described (Gomez-Nicola et al., 2013). (B) Analysis of the density of neural progenitor cells (NPCs; DCX^+^) at the subgranular layer (SGL) of the dentate gyrus (DG) after gain of activity of CSF1R in prion-diseased mice (+CSF1 or +IL34). Quantified data expressed as mean ± SEM of the number of DCX^+^ cells/mm^2^. (C–E) Analysis of the density of proliferating cells (BrdU^+^; D) and NPCs (DCX^+^; E) at the SGL of the DG after loss of activity of CSF1R in prion-diseased mice (+GW2580). Quantified data expressed as mean ± SEM of the number of BrdU^+^ or DCX^+^ cells/mm^2^. Representative images shown in C. (F–H) Fluorescent marking of neuron generation in the DG by the intraparenchymal (hilus) administration of ecotropic SFFV γ-retroviral (Eco-SFFV-RV) vectors driving the expression of mCherry, in ME7 or NBH mice, treated with GW2580 or control (see A). (F) Projection of confocal Z-stacks of mCherry^+^ 4-weeks old granule neurons from NBH (normal brain homogenate) or ME7 mice (see A). The maturation of the dendritic trees was analysed according to the Sholl method (G), representing the mean ± SEM of the number of intersections at increasing distances from the cell soma. (H) Quantification of the maximum distance from cell soma (μm) or the total number of dendrites/neuron, in cells traced as described previously, expressed as mean ± SEM. For G, statistical differences: ^*^p < 0.05 ME7 control vs. NBH control, ^#^*p* < 0.05 ME7 control vs. ME7+GW2580. Data were analysed with a *T*-test. For B, statistical differences: ^*^p < 0.05 (vs. ME7+Sal). For D, E, H, statistical differences for connected bars: ^*^p < 0.05, ^**^p < 0.01. Data were analysed with a one-way (B) or two-way (D, E, H) ANOVA showing statistical comparisons arising from a post-hoc Tukey test (*n* = 4–6). Scale bar in (C) 100 μm, (F) 50 μm.

**Fig. 4:**
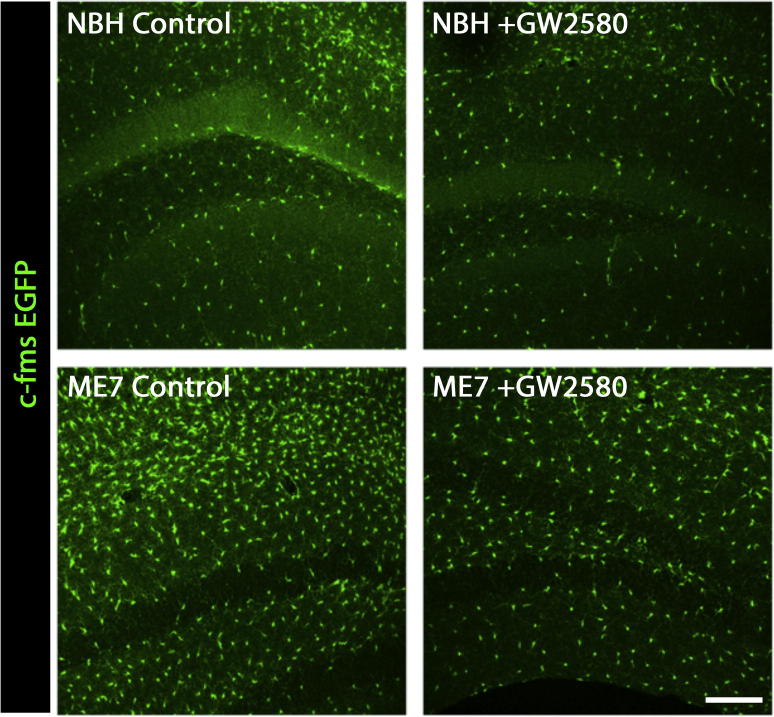
GW2580 inhibits the proliferation of microglial cells in ME7 mice. Analysis of the impact of the long-term treatment (4 weeks) of NBH (normal brain homogenate) or ME7 mice with control or GW2580, on the number of microglial cells. Representative pictures from the DG, with microglial cells identified by the expression of EGFP (macgreen mice; green). Scale bar 200 μm. (For interpretation of the references to colour in this figure legend, the reader is referred to the web version of this article.)

We delivered intracerebral recombinant CSF1 or IL34, ligands for CSFR1, to the hippocampus of prion diseased mice (Fig. 3A) in order to increase microglial proliferation (Gomez-Nicola et al., 2013), and analysed the effects on the number of NPCs (DCX^+^). The increased number of microglia, driven by the activity of both CSF1 and IL34 (Gomez-Nicola et al., 2013), led to an increase in the number of DCX^+^ in the DG (Fig. 3B), suggesting a positive correlation between microglial expansion and neurogenesis. We further investigated this correlation and examined the effects of the inhibition of microglial proliferation by the selective blockade of the tyrosine kinase activity of CSF1R by GW2580 (Gomez-Nicola et al., 2013) in macgreen mice with prion disease (ME7) or NBH controls (Fig. 3A). Oral dosing with GW2580 prevented the amplification of the microglial population in prion disease (Fig. 4), as previously reported (Gomez-Nicola et al., 2013). The analysis of cell proliferation at the neurogenic niche (SGL) showed a significant increase in BrdU^+^ cells in ME7 mice when compared to NBH controls, which was prevented by GW2580 (Fig. 2D), consistent with previously reported data (Gomez-Nicola et al., 2014c). The expansion of DCX^+^NPCs observed in ME7 mice was prevented when microglial proliferation was blocked (Fig. 3C, E), providing evidence that the role of microglia during prion disease was mainly pro-neurogenic.

The observed changes in neurogenic activity upon inhibition of microglial proliferation led us to analyse later stages of the neurogenic cascade. After dosing NBH and ME7 animals with GW2580 we traced newborn neurons with γ-retroviral vectors expressing mCherry, and analysed neuronal differentiation and integration 4 weeks later (Fig. 3A). We observed the characteristic neuronal phenotype in prion diseased granule cells (Gomez-Nicola et al., 2014c): Sholl analysis of the dendritic trees revealed the shorter, hyper-ramified and tortuous dendritic trees of ME7 neurons when compared with NBH neurons (Fig. 3F, G, H). The inhibition of microglial proliferation prevented the defective ramification of granule cells observed in ME7 mice, the granule cells now appear to have morphological parameters similar to those seen in NBH controls (Fig. 3F, G, H).

Our results demonstrate the pro-neurogenic activity of microglia during prion disease, as the inhibition of their proliferation restores baseline levels of neurogenesis. Associated with the reduction in neurogenesis was the notable restoration of a correct neuronal differentiation and a normal dendritic morphology.

### 3.3 Gene expression screening identifies TGFβ as a key regulator of the microglial-dependent pro-neurogenic activity in prion disease

**Fig. 5:**
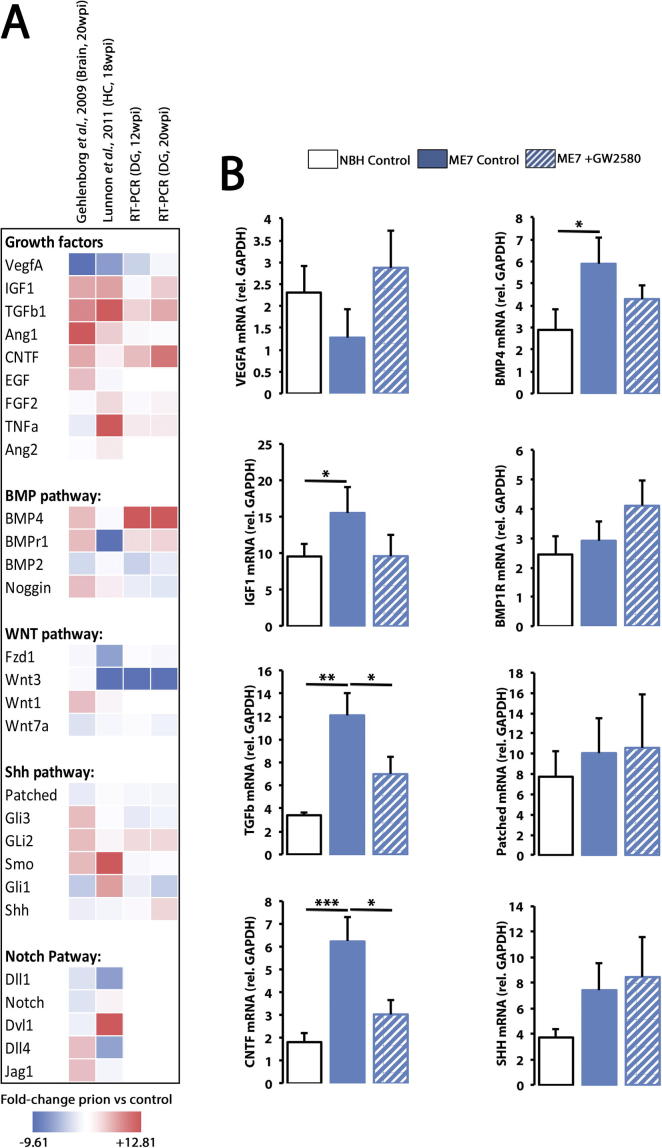
Gene expression screening of potential molecules regulating the pro-neurogenic activity of microglia in prion-diseased mice. (A) Progressive screening of gene expression profiles (see methods and Table S1), from microglia related mRNA species reported in two published microarray studies of models of prion disease, using whole brain (Gehlenborg et al., 2009) or hippocampal samples(Lunnon et al., 2011) and RT-PCR analysis in the dentate gyrus (DG) microdissected samples from both NBH (normal brain homogenate) and ME7 mice, at 12 and 20 weeks post-induction. Data shown colour-coding (blue to red) fold-change of prion vs. control from −9.61 to +12.81. (B) The mRNA expression of shortlisted molecular candidates (*Patched*, *Shh*, *Bmp4*, *Bmp1r*, *Tgfβ1*, *Cntf*, *Igf1*, *Vegfa*) was analysed by RT-PCR in DG microdissected samples from NBH, ME7 and ME7+GW2580 mice. Expression of mRNA represented as mean ± SEM and indicated as relative expression compared to the housekeeping gene (GAPDH) using the 2^−ΔΔCT^ method. Statistical differences for connected bars: ^*^p < 0.05, ^**^p < 0.01, ^***^p < 0.001. Data were analysed with a one-way ANOVA showing statistical comparisons arising from a post-hoc Tukey test (*n* = 4).

We next set out to identify the molecular determinants of the pro-neurogenic actions of microglia in prion disease and designed a progressive screening of gene expression profiles (see methods). We started by analysing the expression levels of microglia related mRNA species reported in two published microarray studies of models of prion disease, using whole brain (Gehlenborg et al., 2009) or hippocampal samples (Lunnon et al., 2011). From a starting list of 28 genes representing the main pathways controlling hippocampal neurogenesis with some link to inflammation (Sup File1), we defined a total of 20 genes to progress to the next step of the screening, selecting those with a clear and consistent trend in the expression profiles (Fig. 5A). The mRNA expression of the 20 candidate genes was analysed by RT-PCR in dentate gyrus microdissected samples from both NBH and ME7 mice, at 12 and 20 weeks post-induction. RT-PCR analysis highlighted 8 candidate genes (*Patched*, *Shh*, *Bmp4*, *Bmp1r*, *Tgfβ1*, *Cntf*, *Igf1*, *Vegfa*) with significant expression alterations in the dentate gyrus of ME7 mice, when comparison to NBH mice (Fig. 5A). The 8 shortlisted genes were analysed in samples from microdissected dentate gyrus NBH, ME7 and ME7+GW2580 mice, in order to pinpoint those associated with microglial cells, as treatment with GW2580 has been shown effective in arresting the amplification of the microglial population in prion disease(Gomez-Nicola et al., 2013). The changes in relative expression of *Tgfβ1*, *Cntf*, *Igf1* and *Bmp4* followed the same directions previously observed for the number of DCX^+^ cells in ME7 mice and ME7 mice in response to GW2580 (Fig. 2E), upregulated in ME7 mice and downregulated when microglial proliferation was blocked (Fig. 5B). These changes suggested that *Tgfβ1*, *Cntf*, *Igf1* and *Bmp4* were likely candidate genes driving the pro-neurogenic actions of microglia in prion disease.

**Fig. 6:**
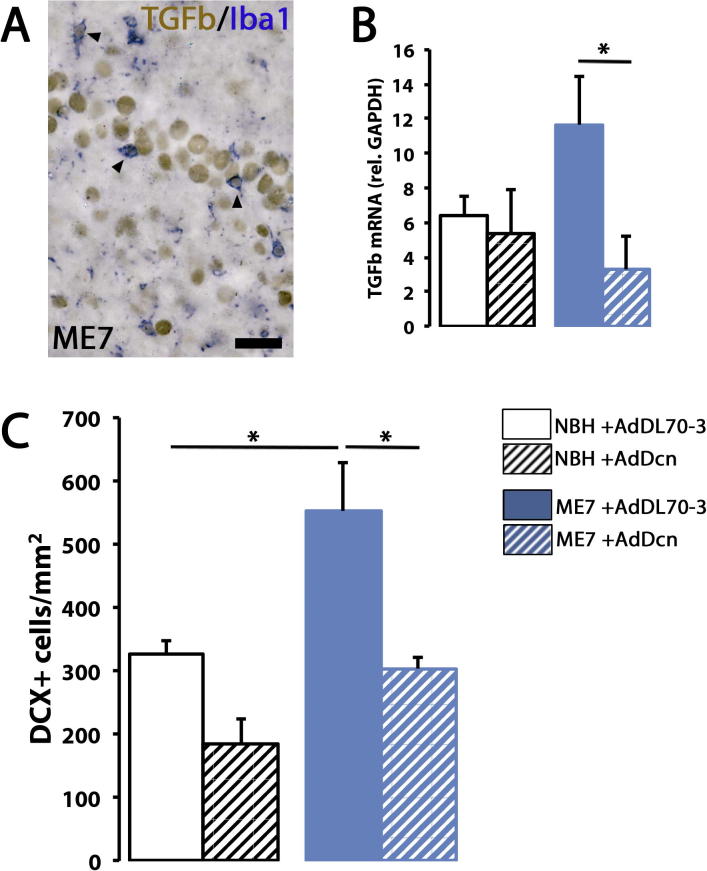
TGFβ is responsible for the microglial pro-neurogenic activity in prion disease. (A) Immunohistochemical analysis of the localisation of TGFβ (brown) in microglial cells (Iba1^+^, blue) in ME7 mice. Representative examples of double-positive cells are indicated by arrowheads. (B) TGFβ mRNA expression analysed by RT-PCR in the hippocampus of NBH+AdDL70-3, NBH+AdDcn, ME7+AdDL70-3 or ME7+AdDcn mice. Expression of mRNA represented as mean ± SEM and indicated as relative expression compared to the housekeeping gene (GAPDH) using the 2^−ΔΔCT^ method. (C) Analysis of the density of neural progenitor cells (NPCs; DCX^+^) at the subgranular layer (SGL) of the dentate gyrus (DG) in NBH+AdDL70-3, NBH+AdDcn, ME7+AdDL70-3 or ME7+AdDcn mice. Quantified data expressed as mean ± SEM of the number of DCX^+^ cells/mm^2^. Scale bar in (A) 50 μm. Statistical differences for connected bars: ^*^p < 0.05. Data were analysed with a two-way ANOVA showing statistical comparisons arising from a post-hoc Tukey test (*n* = 4–6).

We decided to focus in the analysis of the actions of TGFβ, as the actions of this cytokine are key for the function of microglia in health and disease (Boche et al., 2006, Gomez-Nicola and Perry, 2014a, Gomez-Nicola and Perry, 2014b). First, we confirmed TGFβ expression by microglia (Iba1^+^) in ME7 mice by immunostaining (Fig. 6A); we found that a consistent proportion of microglia co-localised with TGFβ in both NBH (61.27%) and ME7 (61.21%) hippocampi (Fig. 6A). To investigate whether TGFβ was the major mediator in the pro-neurogenic actions of microglia, we inhibited the activity of TGFβ by the adenoviral expression of Decorin, a natural inhibitor of TGFβ (Boche et al., 2006). We observed a reduction in TGFβ1 mRNA expression levels in response to the Decorin adenovirus (AdDcn), both in NBH and ME7 mice, when compared with mice injected with a control adenovirus (AdDL70-3) (Fig. 6B). We then analysed the effects of TGFβ inhibition on hippocampal neurogenesis, using the number of DCX^+^ cells as a readout. The inhibition of the activity of TGFβ by AdDcn blocked the disease related expansion of DCX^+^ cells observed in ME7 mice (Fig. 6C). This pro-neurogenic effect of TGFβ in prion disease was also observed (not significant) in NBH mice (Fig. 6C), suggesting a TGFβ -dependent control of baseline neurogenesis. The results observed by the inhibition of TGFβ activity mimicked those observed when preventing the amplification of the microglial population in prion disease, suggesting that TGFβ is a pivotal link between inflammation and neurogenesis.

## 4 Discussion

The study of the impact of neuroinflammation on the regulation of adult neurogenesis is of crucial relevance to understand the potential mechanisms of repair in neurodegenerative diseases. Several chronic neurodegenerative diseases are characterised by a profound innate immune response (Gomez-Nicola and Perry, 2014a, Gomez-Nicola and Perry, 2014b), and aberrant modifications of the neurogenic reserve (Gomez-Nicola et al., 2014c, Miller and Gomez-Nicola, 2014), although how these two processes interact is not yet fully understood. By selectively controlling the microglial number during prion disease, we provide evidence for a pro-neurogenic role of these cells in the adult hippocampal neurogenic niche. We blocked microglial proliferation for 4 weeks using the CSF1R tyrosine kinase inhibitor (GW2580), and demonstrated a reduction in neurogenesis that supports the hypothesis that microglia have a net pro-neurogenic effect during prion disease. These results were corroborated by a gain-of-function experiment, delivering recombinant CSF1 or IL34 and observing the opposite effects namely an increase in neurogenesis. Although a report recently suggested CSF1R is expressed and functions in neurons (Luo et al., 2013), we observed that CSF1R-modulatory approaches are specific for microglia, and found no evidence that the receptor is expressed either by neurons or astrocytes, supporting previously reported data (Erblich et al., 2011, Gomez-Nicola et al., 2013, Sasmono et al., 2003).

The present data progress recently published data from our group, reporting an increase in neurogenesis during prion disease that counteracts the extensive neuronal loss in the dentate gyrus (Gomez-Nicola et al., 2014c). However, the neurons newly generated during prion disease fail to develop a normal differentiation pattern and to regenerate morphology or electrophysiological properties of the lost granule cells, supporting the hypothesis that although prion disease induces a compensatory self-repairing mechanism this fails to restore the lost functions (Gomez-Nicola et al., 2014c, Miller and Gomez-Nicola, 2014). We have demonstrated that some factors derived from microglia contribute to the aberrant increase in neurogenesis, and also play a role in the abnormal development and differentiation of newborn neurons as evidenced by the restoration of a normal differentiation program when microglial proliferation was inhibited. Although microglia would unlikely determine all the biology of NSCs in this model, these complementary actions of microglia suggest that the pharmacological strategies targeting microglial proliferation in chronic neurodegeneration could be beneficial to prevent the aberrant neurogenic events observed not only in prion disease, but also in other chronic neurodegenerative diseases (Gomez-Nicola et al., 2014c, Li et al., 2009, Llorens-Martin et al., 2013, Sun et al., 2009).

Previous studies are in line with our findings as they show the important contribution of microglia for the maintenance and support of hippocampal neurogenesis in the steady state and during pathological events (Butovsky et al., 2006, Ekdahl et al., 2009, Ziv et al., 2006). For example, microglia can have a stimulating effect by providing soluble factors essential for neurogenesis (Aarum et al., 2003, Walton et al., 2006) although other reports support the hypothesis that microglial expansion is detrimental for hippocampal adult neurogenesis (Kempermann and Neumann, 2003). Monje *et al.* reported a decrease in neurogenesis *in vitro* when precursor cells were exposed to a conditioned medium from activated microglia (Monje et al., 2003). Ekdahl *et al.* demonstrated that LPS-induced microglia alters neurogenesis and that suppression of the microglial response by minocycline, an anti-inflammatory drug, restores the neurogenic activity of the hippocampus that was impaired by inflammatory processes (Ekdahl et al., 2003). Further research supports the idea that whether the effect of activated microglia in the injured CNS is positive or negative is determined by the microenvironment changes which tightly regulate the state of activation and the functional phenotype of microglia (Butovsky et al., 2006, Gomez-Nicola and Perry, 2014b). More importantly, recent studies reported that a dampened microglial response during chronic unpredictable stress caused a decline in neurogenesis, which restored after the stimulation of microglial proliferation by CSF1 (Kreisel et al., 2014).

The cytokine profile of prion disease is dominated by high levels of TGFβ suggesting a key role of this cytokine in neurodegenerative disorders (Boche et al., 2006). TGFβ is known to act as an anti-inflammatory and neuroprotective agent (Flanders et al., 1998). In this study, using a screening of potential microglial-derived modulators of neurogenesis, we highlight the relevance of TGFβ in controlling the aberrant pro-neurogenic activity observed in prion disease. We modulated TGFβ activity using an adenovirus to deliver a natural inhibitor of TGFβ, Decorin (AdDcn), and provide mechanistic evidence for the pro-neurogenic role of TGFβ. A previous study demonstrated an increase of TGFβ expression throughout the progression of prion disease, which coincides with the progressive enhanced microglial activation observed during this time course (Boche et al., 2006). The parallel evolution of these two features suggests a strong link between microglia and TGFβ during prion disease, evidenced now by our data resulting from the inhibition of microglial proliferation. In addition to the reported effects of microglial proliferation on neurogenesis in prion diseased mice, we noted a trend supporting a TGFβ-dependent pro-neurogenic effect of microglia in control mice. NBH mice show the same balance of microglia/DCX^+^ cells compared to ME7 mice and the same proportion of the population express TGFβ as ME7 mice, suggesting a similar regulatory system being in place for controlling neurogenesis in a TGFβ-dependent manner. One key factor limiting the impact of the anti-proliferative strategies on NBH mice is the very low rate of proliferation of microglia in the normal brain (Lawson et al., 1992). To overcome these difficulties, we predict that a sustained and long-term usage of CSF1R inhibitors in normal mice would provide a useful approach to study the role of microglia in neurogenesis in the normal healthy brain.

The observation that TGFβ has pro-neurogenic actions in the healthy and diseased neurogenic niche would be consistent with recently reported data on ALK5 (TGFβ Receptor I), which suggests that the activation of TGFβ signaling has proneurogenic functions in the healthy hippocampal neurogenic niche (He et al., 2014), as suggested by previous studies (Battista et al., 2006, Mathieu et al., 2010). However, TGFβ functions in the neurogenic niche are a subject of controversy. TGFβ has been shown to induce a negative feedback regulation in controlling the NSCs pool (Wachs et al., 2006). The apparent controversy between these studies and our data could be explained by a context-dependent (healthy brain vs. diseased, hippocampus vs. subventricular zone) mode of action of TGFβ, as well as cell-dependent functions as evidenced here by the microglia-derived TGFβ. For example, serum-derived factors are able to determine the effect of TGFβ on the neurogenic niche (Kane et al., 1996, Wachs et al., 2006). Activated microglia have been identified as one of the cells that contributes to TGFβ production, with microglial TGFβ expression increased in response to cerebral ischemia and leading to enhanced neuronal survival (Flanders et al., 1998). In addition, TGFβ inhibits macrophage function by regulating the production of pro-inflammatory cytokines, effect potentiated when cooperating with CSF1, suggesting a strong interaction of microglial proliferation and TGFβ signaling (Lodge and Sriram, 1996). These ideas suggest that a more targeted analysis of the interaction of TGFβ with different cells is needed, in order to better understand the multiple roles of this cytokine *in vivo*.

Our gene screening strategy highlighted other microglia-derived molecules potentially regulating hippocampal neurogenesis. We found changes in the BMP pathway that could explain the progressive decrease in hippocampal neurogenesis previously reported in prion diseased mice (Gomez-Nicola et al., 2014c). The role of the BMP pathway in adult neurogenesis has not been fully determined, however, there is evidence to suggest a role in driving NSCs to quiescence. The activation of BMPR1A determines the rate of neurogenesis and prevents NSCs pool depletion, a system that can be inhibited by Noggin (Mira et al., 2010). This hypothesis is supported by our results that show an increase in BMP4 and BMP1RA mRNA, as well as a decrease in Noggin mRNA, during the disease. The observed inhibition of this pathway correlates with the late decline in neurogenesis observed in ME7 mice, after a pro-neurogenic surge that extends until mid-late phases of the model (Gomez-Nicola et al., 2014c). This suggests that BMP4 may be a microglial-derived factor, while BMP1RA is not, consistent with previously reported findings on this receptor (Mira et al., 2010). While BMP4 expression has been reported in oligodendrocytes and astrocytes (Sabo et al., 2011), no studies have been directed to determine its functions in microglia.

The observed expression trends of CNTF and IGF1 may also be of interest when considering microglia-derived factors regulating hippocampal neurogenesis. Both molecules have roles in providing neurotrophic support to nascent neurons (Aberg et al., 2000, Sendtner et al., 1994). CNTF is responsible for regulating the size of the NSCs pool and their differentiation. Experiments with CNTF^-/-^ mice and anti-CNTF antibodies showed reductions in proliferation and number of NSCs and newborn neurons (Muller et al., 2009) whereas CNTF infusion in the forebrain increased them (Bath and Lee, 2010), thereby suggesting an essential role in maintaining neurogenic activity. Our results emphasise these findings and provide new evidence of its role during disease; an increase in CNTF during the late stage of ME7 may be a way of opposing the ongoing neurodegeneration by providing trophic support to forming neurons. Although this mechanism appears to be microglia-dependent, CNTF expression has been reported so far only in Schwann cells and astrocytes (Ekdahl et al., 2003) and its expression and activity in microglia still needs to be studied. A similar trend is observed for IGF1 expression, which is upregulated in prion disease and appears to be microglia associated. Circulating IGF1 has neurotrophic and neurogenic functions (Aberg et al., 2000, Trejo et al., 2001) and the neuroprotective actions of IGF1 are key for chronic neurodegenerative diseases (Carro et al., 2002), suggesting a link between microglia and IGF1 expression.

In summary, by taking advantage of the selective targeting of microglial proliferation, we have uncovered a pro-neurogenic role for microglia in chronic neurodegeneration. Microglia exert this pro-neurogenic role as least in part through TGFβ, linking a key neuroinflammatory pathway with the intrinsic self-repairing potential of the brain, which opens new avenues for the design of neuroprotective and restorative approaches.

## Competing financial interests

The authors have no competing financial interests.

## Appendix A Supplementary data

Supplementary Table S1 Summary of the functions of the main candidate mitogens/pathways regulating hippocampal neurogenesis. Literature was searched for genes involved in neurogenesis and inflammation, showing their overall neurogenic effect, proposed mechanism of action and reference.

Supplementary Table S2 RT-PCR primers used in this study.
