# Supplementary material for: Microglia regulate hippocampal neurogenesis during chronic neurodegeneration
Source: Brain Behav Immun. 2016 Jul;55:179–90. doi: 10.1016/j.bbi.2015.11.001 (PMC4907582; doi:10.1016/j.bbi.2015.11.001)
Supplement: Supplementary Table S1 — Summary of the functions of the main candidate mitogens/pathways regulating hippocampal neurogenesis. Literature was searched for genes involved in neurogenesis and inflammation, showing their overall neurogenic effect, proposed mechanism of action and reference. [file mmc1.docx]

| **Mitogen/**  **Pathway** | **Reference** | **Neurogenic**  **effect** | **Comments** |
| --- | --- | --- | --- |
| VEGFA | Schänzer *et al*., 2004 | **+** | Reduces apoptosis resulting in NSCs expansion due to increased survival. |
| IGF1 | Erickson *et al*., 2008 | + | Promotes NSCs proliferation and differentiation via interactions with the ERK and Akt pathways. Shown to expand neurosphere size *in vitro*. |
| TGFβ1 | Kandasami *et al*., [2010](http://journals.lww.com/jneuropath/toc/2010/07000)  Wachs *et al*., 2006  Buckwalter *et a*l., 2006  He *et al.,* 2014  Battista *et al.,* 2006 | **- / +** | Impairs proliferation by arresting the cell cycle of neural precursors and stem cells in G0/1 phase. Promotes stem cell quiescence. Does not affect differentiation.  Also shown to increase adult neurogenesis both in vivo and in primary cultures, by increasing proliferation. Furthermore, deletion of the TGF beta receptor caused fewer and less arborized newborn neurons whilst also impairing their migration. |
| ANG1 | Bai *et al*., 2009 | **+** | Promotes differentiation and neurite outgrowth. |
| ANG2 | Liu *et al*., 2009 | **+** | Promotes differentiation into neurons and regulates NPCs migration |
| CNTF | Blanchard J *et al*., 2010  Kang *et al.,* 2012 | **+** | Enhances proliferation by inducing FGF2 expression. Induces early neuronal fate as well as differentiation and improved survival of new neurons. |
| EGF | Doetsch *et al*., 2009 | **+** | Enhances proliferation of transient-amplifying cells. |
| FGF2 | Werner *et al*., 2001 | **+** | Aids maturation of new neurons. |
| TNFα | Widera *et al*., 2009 | **+** | Increases proliferation by activating NF-kB signaling |
| BMP pathway | Mira *et al*., 2010 | **-** | Decreases proliferation and inhibits differentiation. |
| WNT pathway | L'Episcopo *et al*., 2012  Hussaini *et al*., 2014 | **+** | Regulates neuronal maturation and maintenance of the progenitor pool. |
| SHH pathway | Yaddanapudi *et al*., 2011 | **+** | Upregulates NSCs proliferation. |
| Notch Pathway | Dave *et al*., 2011  Cao *et al*., 2011 | **+/-** | Increases proliferation and inhibits neural fate. |

**Table S1. Summary of the functions of the main candidate mitogens/pathways regulating hippocampal neurogenesis.** Literature was searched for genes involved in neurogenesis and inflammation, showing their overall neurogenic effect, proposed mechanism of action and reference.

1. Schänzer, A. *et al.* Direct Stimulation of Adult Neural Stem Cells In Vitro and Neurogenesis In Vivo by Vascular Endothelial Growth Factor. *Brain Pathol.* **14,** 237–248 (2006).

2. Erickson, R. I., Paucar, A. A., Jackson, R. L., Visnyei, K. & Kornblum, H. Roles of insulin and transferrin in neural progenitor survival and proliferation. *J. Neurosci. Res.* **86,** 1884–94 (2008).

3. Kandasamy, M. *et al.* Stem Cell Quiescence in the Hippocampal Neurogenic Niche Is Associated With Elevated Transforming Growth Factor-β Signaling in an Animal Model of Huntington Disease. *J. Neuropathol. Exp. Neurol.* **69,** 717–728 (2010).

4. Wachs, F.-P. *et al.* Transforming growth factor-beta1 is a negative modulator of adult neurogenesis. *J. Neuropathol. Exp. Neurol.* **65,** 358–70 (2006).

5. Buckwalter, M. S. *et al.* Chronically increased transforming growth factor-beta1 strongly inhibits hippocampal neurogenesis in aged mice. *Am. J. Pathol.* **169,** 154–64 (2006).

6. He, Y. *et al.* ALK5-dependent TGF-β signaling is a major determinant of late-stage adult neurogenesis. *Nat. Neurosci.* **17,** 943–952 (2014).

7. Battista, D., Ferrari, C. C., Gage, F. H. & Pitossi, F. J. Neurogenic niche modulation by activated microglia: transforming growth factor β increases neurogenesis in the adult dentate gyrus. *Eur. J. Neurosci.* **23,** 83–93 (2006).

8. Bai, Y. *et al.* Ectopic expression of angiopoietin-1 promotes neuronal differentiation in neural progenitor cells through the Akt pathway. *Biochem. Biophys. Res. Commun.* **378,** 296–301 (2009).

9. Liu, X. S. *et al.* Angiopoietin 2 mediates the differentiation and migration of neural progenitor cells in the subventricular zone after stroke. *J. Biol. Chem.* **284,** 22680–9 (2009).

10. Blanchard, J. *et al.* Beneficial effect of a CNTF tetrapeptide on adult hippocampal neurogenesis, neuronal plasticity, and spatial memory in mice. *J. Alzheimers. Dis.* **21,** 1185–95 (2010).

11. Kang, S. S. *et al.* Endogenous CNTF mediates stroke-induced adult CNS neurogenesis in mice. *Neurobiol. Dis.* **49,** 68–78 (2013).

12. Doetsch, F., Petreanu, L., Caille, I., Garcia-Verdugo, J. M. & Alvarez-Buylla, A. EGF converts transit-amplifying neurogenic precursors in the adult brain into multipotent stem cells. *Neuron* **36,** 1021–34 (2002).

13. Werner, S., Unsicker, K. & von Bohlen und Halbach, O. Fibroblast growth factor-2 deficiency causes defects in adult hippocampal neurogenesis, which are not rescued by exogenous fibroblast growth factor-2. *J. Neurosci. Res.* **89,** 1605–17 (2011).

14. Widera, D., Mikenberg, I., Elvers, M., Kaltschmidt, C. & Kaltschmidt, B. Tumor necrosis factor alpha triggers proliferation of adult neural stem cells via IKK/NF-kappaB signaling. *BMC Neurosci.* **7,** 64 (2006).

15. Mira, H. *et al.* Signaling through BMPR-IA regulates quiescence and long-term activity of neural stem cells in the adult hippocampus. *Cell Stem Cell* **7,** 78–89 (2010).

16. L’Episcopo, F. *et al.* Plasticity of subventricular zone neuroprogenitors in MPTP (1-methyl-4-phenyl-1,2,3,6-tetrahydropyridine) mouse model of Parkinson’s disease involves cross talk between inflammatory and Wnt/β-catenin signaling pathways: functional consequences for neuropr. *J. Neurosci.* **32,** 2062–85 (2012).

17. Hussaini, S. M. Q. *et al.* Wnt signaling in neuropsychiatric disorders: ties with adult hippocampal neurogenesis and behavior. *Neurosci. Biobehav. Rev.* **47,** 369–83 (2014).

18. Yaddanapudi, K., De Miranda, J., Hornig, M. & Lipkin, W. I. Toll-like receptor 3 regulates neural stem cell proliferation by modulating the Sonic Hedgehog pathway. *PLoS One* **6,** e26766 (2011).

19. Dave, R. K. *et al.* Sonic hedgehog and notch signaling can cooperate to regulate neurogenic divisions of neocortical progenitors. *PLoS One* **6,** e14680 (2011).

20. Cao, Q., Kaur, C., Wu, C.-Y., Lu, J. & Ling, E.-A. Nuclear factor-kappa β regulates Notch signaling in production of proinflammatory cytokines and nitric oxide in murine BV-2 microglial cells. *Neuroscience* **192,** 140–54 (2011).
