# Supplementary material for: Microglia regulate hippocampal neurogenesis during chronic neurodegeneration
Source: Brain Behav Immun. 2016 Jul;55:179–90. doi: 10.1016/j.bbi.2015.11.001 (PMC4907582; doi:10.1016/j.bbi.2015.11.001)
Supplement: Supplementary Table S2 — RT-PCR primers used in this study. [file mmc2.docx]

| **Gene** | **ID** | **FW (5’-3’)** | **RW (5’-3’)** |
| --- | --- | --- | --- |
| **VEGFA** | NM_001287056 | GCAGCGACAAGGCAGACTAT | AACCTCCTCAAACCGTTGGC |
| **IGF1** | NM_010512 | AGATCTGCCTCTGTGACTTCTTGA | AGCCTGTGGGCTTGTTGAAGT |
| **TGFb1** | NM_011577 | TGTACGGCAGTGGCTGAACC | CGTTTGGGGCTGATCCCGTT |
| **ANG1** | NM_009640 | GCCCAAAGCCATCAGCAATC | GGTTGCACATCCAAGCCAAG |
| **CNTF** | NM_170786 | CCTTCACCGCCGGGACCTCT | TCCAGCGATCAGTGCTTGCCAC |
| **FGF2** | NM_008006 | ACCCACACGTCAAACTACAAC | TAACACACTTAGAAGCCAGCA |
| **TNFa** | NM_013693 | AGGCACTCCCCCAAAAGATG | TTGCTACGACGTGGGCTAC |
| **BMP4** | NM_007554 | TGGTCTCCGTCCCTGATGGG | CGGAATGGCTCCATTGGTTCCT |
| **BMPR1a** | NM_009758 | AGGGTGGGCACCAAGCGGTA | GGCCGCAAGCGTTTCACACA |
| **BMP2** | NM_007553 | TGGACGTGCCCCCTAGTGCT | GGATGCCGCGGCGAACTTCT |
| **Noggin** | NM_008711 | CGGCGGCCAGCACTATCTAC | TCAGGGGGCGAAGTAGCCAT |
| **Frizzled1** | NM_021457 | CCTTTGCGCTGTGAAGCGGG | GAGCGCCCAACTCTCGGCAG |
| **Wnt3a** | NM_009522 | CCTGTTCTGGACAAAGCCACCC | GGCTGCTGCACCCACAGATA |
| **Wnt7a** | NM_009527 | GCGCAGGCTATGTGGATTGC | CACCGAAGAGAAGCCACCGA |
| **Patched1** | NM_008957 | GACCGGCCTTGCCTCAACCC | GGGCGTGAGCGCTGACAAGT |
| **Gli3** | NM_008130 | AGAGCAAGCCCACAAGCGAG | TTCCTTCCGGCTGTTCCTGC |
| **Gli2** | NM_001081125 | TCCACGCGCCTTTGCCGATT | GGTGGGCGCTGAAGGGTGAC |
| **Smothened** | NM_176996 | GAAGGCTGCCCAAACGAGGT | GAATCCCACAGCCCTCCACG |
| **Gli1** | NM_010296 | CCCCGACGGAGGTCTCTTTG | GGCCGTCCCAACTGCTTCTT |
| **SHH** | NM_009170 | CGGCTGATGACTCAGAGGTGC | CGGTCACTCGCAGCTTCACT |
| **GAPDH** | NM_008084.2 | TGAACGGGAAGCTCACTGG | TCCACCACCCTGTTGCTGTA |

**Table S2**. RT-PCR primers used in this study.
